# Crop residue burning increased during the COVID-19 lockdown: A case study of rural India

**DOI:** 10.1016/j.heliyon.2024.e27910

**Published:** 2024-03-09

**Authors:** Adrian A. Lopes, Ajalavat Viriyavipart

**Affiliations:** Department of Economics, School of Business Administration, American University of Sharjah, United Arab Emirates

**Keywords:** Crop residue burning, COVID-19 lockdown, Unintentional consequences, Farmland management

## Abstract

The customary practice of crop residue burning (CRB) is a major policy concern across several developing economies because of the associated increase in air pollution and reduction in soil quality. CRB poses a hazard to public health and sustainable farmland management. We collected original survey data from a panel of 400 wheat farmers on CRB choices during April–May of 2019 and 2020 – with the latter coinciding with India's COVID-19 nationwide lockdown. This timeline of events facilitated a unique identification of changes in CRB that are attributable to the lockdown. Several studies find that lockdowns during 2020 had beneficial effects on the environment owing to reduced economic activity. However, our findings indicate that CRB may have unintentionally increased during the lockdown. A binary variable regression framework analyzes the determinants of CRB choices of farmers over two years. We control for farmers' opinions on various socioeconomic aspects of the pandemic lockdown to examine its effects on their CRB decisions. The lockdown significantly increased the likelihood of CRB by up to 12%. Furthermore, farmers who lost agricultural income and those compelled to sell assets during the lockdown were 22% and 19% more inclined, respectively, to choose CRB. Labor mobility ceased during the lockdown and increased the cost of environmentally friendly farmland management; this increased the likelihood of CRB by 6%. This study contributes to a growing literature on the unintentional consequences of pandemic lockdowns.

## 1 Introduction

The postharvest burning of agricultural stubble or crop residue is a major policy concern for public health, agriculture, and environmental sustainability in several developing economies. Each year, countries like India, China, Egypt, Pakistan, and Thailand witness increases in air pollution from postharvest crop residue burning (CRB) [[1], [2], [3], [4], [5]]. The smoke from CRB spreads to neighboring rural towns and urban centers [[6], [7], [8]] and leads to increases in respiratory illnesses [9,10]. Moreover, CRB lowers farmland quality by destroying the organic matter within soil ecosystems [11,12]. In the context of the COVID-19 pandemic, there has been a spate of recent studies examining the effects of lockdowns on the natural environment. Several of these studies have inferred that the sudden disruptions to economic activity yielded environmental benefits [[13], [14], [15], [16], [17], [18], [19], [20], [21], [22], [23], [24]]. While in many instances, the lockdowns may have been immediately beneficial for the environment, it is important to consider plausible occurrences of the opposite and examine its reasons. Some studies have brought to the fore an array of unintended negative environmental consequences of COVID-19 lockdowns [[25], [26], [27], [28]]. In this paper, we consider a case study of a rural region in northern India where CRB likely increased during the nationwide lockdown imposed in 2020. We shall also discuss the literature on both the positive and negative environmental effects of COVID-19 lockdowns and how this paper contributes to its growing narrative. Presently, there is little consensus on whether COVID-19 lockdowns were beneficial to the environment or not, thus making it an important issue to investigate further.

In India, burning agricultural stubble is considered illegal, and the National Green Tribunal governmental body recommends that fines be imposed upon errant farmers [29]. It is conceivable that illegal economic activity may have increased in the complete absence of monitoring and enforcement during lockdown periods around the world. The sudden disruption to economic activity and the lack of monitoring of CRB occurrences warrants a closer examination of plausible effects that the lockdown might have had on CRB in India. We collected original data from a panel of 400 wheat farmers in the Karnal district of Haryana state and recorded their wheat stubble management choices (including burning and alternative methods) during April–May 2019 and 2020. A strict COVID-19 lockdown was enforced across India in phases from the end of March 2020 to the end of May 2020 [17]. The second half of the lockdown coincided with the wheat harvest season in northern India's states. This unique setting provides a pertinent time frame in which to identify the effect that the lockdown may have had on postharvest crop residue management. Migrant farm labor is employed not only for harvesting crops but also for postharvest residue clearing of farms [30]. Employment of farm labor typically witnesses a discrete jump during the harvest season in India. However, one consequence of India's nationwide lockdown was a severe shortage of labor since seasonal farmhands were prohibited from traveling across districts and states [31]. Consequently, our findings point to a significant increase in CRB during 2020; this is largely attributable to severe shortages of farm labor during the nationwide lockdown.

Rice and wheat are the two major crops grown in Haryana, Punjab, and Uttar Pradesh. Rice is harvested in the winter, followed by wheat in the summer. Farmers choose stubble burning because of shortages in farm labor [32,33], and it is a quick and inexpensive method to prepare fields for subsequent cropping cycles [4,[34], [35], [36], [37], [38]]. One notable aspect is that CRB is generally more widespread in rice residue management as opposed to in wheat residue because the latter can be used for cattle fodder [39] while the former cannot.^1^ Consequently, the issue of wheat stubble burning has drawn much less attention in the literature as compared to rice residue burning [41]. Thereby, it is interesting to note that the increase in CRB in our case study occurred after the wheat harvest– a crop whose residue is not typically burned because of its positive tradable value as cattle fodder.

We use original survey data to investigate the incremental impact that the nationwide lockdown had on the availability of hired farm labor and the extent to which it may have led to an increase in wheat residue burning in Haryana. We conducted a phone interview among 400 farmers across eight villages in the Karnal district of Haryana. These farmers form a subset of an original survey of 1230 farmers that we conducted in 2018 (reported in Ref. [4]). We recorded these farmers’ wheat residue management practices in April–May 2019 (one year before the lockdown) and April–May 2020 (during the lockdown). We find nearly a 36 percent increase in postharvest wheat residue burning during the 2020 lockdown compared to the corresponding season of 2019.

Among farmers who chose CRB in 2020, more than 90 percent responded that the unavailability (shortage) of hired migrant labor and its consequent high cost are the main reasons for doing so. More than half of them explicitly stated that residue burning was associated with the pandemic lockdown. We use a logit regression framework to further investigate which attributes of the pandemic increase the likelihood of CRB among farmers who had not used CRB in 2019. We show that two attributes of the pandemic – farmers who had lost agricultural income and farmers who had to sell assets – along with farmers who faced a delay in residue removal, were more likely to use CRB. The responses to these attributes provide a unique insight into farmers’ decision-making processes in the context of the pandemic.

This paper is organized into the following sections. We discuss the literature review in Section 2. In Section 3, we describe our data collection and regression method. In Section 4, we examine the farmers' wheat residue management choices, the reasons for deciding to burn residue, and alternative methods of agricultural postharvest production management. In Section 5, we use a regression framework to analyze the effects that the COVID-19 lockdown had on farmers’ decisions regarding wheat CRB. Section 6 concludes with a discussion of the salient findings in the context of the literature and their plausible implications for policy design in the event of future lockdowns.

## 2 Literature review

This study analyzes how the nationwide lockdown in India affected the farmers’ decisions to burn wheat residue instead of relying on traditional or alternative residue management options. While a developing body of literature suggests that lockdowns were associated with beneficial environmental effects, we aim to contribute to an emerging narrative on the likelihood of their unintentional and counterintuitive environmental effects. In this paper, we explore this subject matter using the lens of management choices of pertinent agricultural stakeholders.

Several studies have found evidence of positive environmental consequences of COVID-19 lockdowns. For instance, Xu et al. [21] show that the average ambient air concentrations of PM2.5, PM10, SO_2_, CO, and NO_2_ were substantially lower in multiple Chinese cities during February 2020 when lockdowns were imposed. They examine air quality data across three cities in central China and find largely positive effects of lockdowns on reducing these pollutants in 2020 as compared to between January and March 2017 through 2019. In another study of pollution levels in five major cities in India, Ravindra et al. [18] examine PM2.5 data during three phases: pre-lockdown in March 2020, lockdown in May 2020, and post-lockdown in July–August 2020. They also find that particulate matter levels were substantially lower during the lockdown phase. In another study, Wang & Su [24] use satellite data from China during the lockdown to find substantial reductions in nitrogen dioxide. Owing to the lockdown, their findings signify a short-term improvement in China's air quality alongside reductions in carbon emissions. Subsequently, Wang & Zhang [42] find that China witnessed an economic recovery and increased energy consumption with an easing of lockdowns. This points to how lockdowns improved environmental quality in general. While governments around the world implemented policies to help with economic recovery [43], the lifting of lockdowns was associated with declining environmental quality [42].

Wang & Huang [22] discuss the importance of examining the impact of the pandemic on the environment, economy, public health, and sustainable development goals. A bulk of this research has focused more on developed countries mainly because of data availability. However, the less developed and developing countries are more susceptible to environmental and health shocks, and thus studying their effects in such countries is important from both policy and sustainability perspectives. Wang & Huang [22] find that the pandemic had induced adverse effects on several of the sustainable development goals and environmental sustainability. They highlight the need for more such studies to systematically understand the effects of the pandemic in less developed and developing countries.

Other studies and reports have paid attention to the presence of negative and/or counterintuitive environmental consequences of the COVID-19 pandemic and its lockdowns. For instance, Shakoor et al. [27] examine variations of environmental pollutants before and during the lockdown periods in the US during the first quarters of 2019 and 2020. They show that while carbon monoxide, nitrogen dioxide, and PM2.5 levels decreased, PM10 and sulfur dioxide unexpectedly increased across five US states during the lockdown. Wang et al. [44] unexpectedly found that in Hangzhou, China, there was an increase in ozone levels in urban and rural areas, and of sulfur dioxide in rural areas during the lockdown. The rural areas witnessed this increase in sulfur dioxide because of increased coal use for heating and from fireworks during the spring eve and lantern festivals, which were celebrated during lockdown. In another study, Rawat & Naja [28] examine carbon monoxide, nitrogen dioxide, and ozone levels at several locations and atmospheric altitudes in India and find evidence of increases in all these pollutants. They report how their findings contradict those of other surface-based pollutant measurement studies, which showed lower air pollution during the lockdown phase in India.

Yang et al. [25] examine the environmental effects of COVID-19 across 25 countries in different continents. They find both positive and negative aspects that merit consideration from an environmental policy standpoint. While their review indicates improvements in particulate matter, nitrogen dioxide, and carbon emission levels, they also note that sulfur dioxide and ozone levels either increased or did not change significantly. They also report on improvements in surface, coastal, and groundwater quality, but not groundwater reservoirs. Furthermore, they report on findings of large quantities of COVID-19 medical and protective equipment waste generation, which increased substantially during lockdowns across the world. An accumulation of solid waste gave way to increases in soil contamination. This was caused by large amounts of uncollected household waste and the disposal of medical protective equipment, face masks, and their packaging.

In another paper, Wang & Li [23] find mixed evidence of the effects of the pandemic on pollutants across several cities and countries. While nitrogen dioxide and particulate matter generally witnessed declines during lockdowns, ozone tended to increase slowly, and trends in sulfur dioxide were not discernible. On a similar note, even though Xu et al. [21] find positive impacts of lockdowns on reducing particulate matter and nitrogen dioxide, they also find evidence of increases in ozone levels. Other examples of negative environmental effects of lockdowns include reports of how illegal harvesting and poaching of endangered animal species increased during COVID-19 lockdowns in countries such as Kenya and Cambodia [26]. The increase in illegal activity is attributable to the sudden reduction in monitoring and law enforcement during lockdown periods.

Although there are studies that find evidence of positive environmental consequences of COVID-19 lockdowns, there also exist other studies that find evidence of the opposite or negative consequences. Clearly, without a consensus that COVID-19 lockdowns were entirely beneficial to the environment, it becomes even more imperative to dig deeper into the matter. The case study explored in this paper highlights some unintentional negative consequences of COVID-19 lockdowns on the environment and contributes towards a growing body of literature on this subject. The empirical data analyzed in this paper utilizes a novel setting of a pertinent time frame to identify the effect of a COVID-19 lockdown on CRB in India by contrasting farmers' choices in two consecutive wheat harvesting seasons – one in 2019 and the other during lockdown in 2020. This approach of contrasting pre-lockdown and during-lockdown effects is like some other papers referenced above. However, we provide an additional and novel perspective on this matter by also accounting for farmers’ subjective opinions on various socioeconomic attributes of the COVID-19 lockdown and examining their impact on CRB decisions. We have also accounted for the shock to labor mobility during the lockdown that led to environmentally friendly farmland management options becoming substantially more expensive. This paper contributes towards the narrative of studies examining the effects of the pandemic in the developing world, which is a growing need with both regional and global significance for environmental policy and sustainability [22].

## 3 Data collection and regression method

### 3.1 Data collection

Our study area lies in the district of Karnal, Haryana state, in northern India. We collected data via a phone survey as the lockdown restrictions did not permit us to conduct face-to-face interviews in the field. We designed and implemented the phone survey in October 2020 with 50 randomly chosen farmers from each of the eight villages for a total of 400 farmers.^2^ This sample derives from a subset of an earlier study of ours [4] in which we recorded and examined the postharvest paddy (rice) management practices of 1230 farmers across twelve villages in the same district of Haryana in the year 2018.^3^ We randomly selected the farmers while ensuring that subject representation was proportionate to Ref. [4] for each village regarding the response to the question “In the last 5 years, was paddy residue ever burnt on your land?” in that survey. For the subsample of 400 farmers for the present study, we recorded their postharvest wheat residue management practices in April–May of the two subsequent years, 2019 and 2020, i.e., before and during lockdown in 2019 and 2020, respectively.

The survey questionnaire (see Appendix) was developed with two broad sections. Section 1 collates information on demographic and socioeconomic characteristics of the farmers including – gender, age, education, occupation, and cultivated acreage for wheat and other crops. Section 2 of the questionnaire focuses on choices of wheat residue management in 2019 and 2020, the reasons for their choices, alternative methods to manage wheat residue, and other questions related to how the COVID-19 pandemic and lockdown influenced their crop residue management decisions. The survey was designed using the questionnaire template of our previous paper on rice residue burning [4] with modifications made for the context of wheat residue management. Specifically, we included questions on alternatives for wheat residue-such as usage for cattle fodder, which was not the case for rice residue. Moreover, this survey had a significant focus on COVID-19 lockdown-related aspects – such as what effects the lockdown had on residue management and recording farmers’ subjective opinions on various socioeconomic attributes of the lockdown. The survey also included questions on how labor mobility was affected during the lockdown and whether it affected farm labor employment for residue management. The questionnaire had been pre-tested with a pilot of eight farmers before its final version.

The questionnaire was translated from English to the local Hindi dialect. The survey study was approved by the Institutional Review Board at the American University of Sharjah (reference #20–010). Furthermore, a detailed information session about the research objectives and activities was held with the various village heads (*sarpanch*) before the survey commenced. Each survey respondent was either the household head or the household's principal decision-maker for agricultural management choices.

### 3.2 Regression framework

The central hypothesis to be explored is that an increase in CRB is attributable to the COVID-19 lockdown, which coincided with the 2020 postharvest wheat season. To investigate the factors that influence a farmer's decision to choose CRB, we estimate a logit regression model given by Equation [1].

(1) $$P\left(CRB\right)=\frac{\exp\left(\beta_{0}+\sum_{n=1}^{N}\beta_{n}X_{n}\right)}{1+\exp\left(\beta_{0}+\sum_{n=1}^{N}\beta_{n}X_{n}\right)}$$

where P(CRB) is the probability that the farmer chose CRB in 2020 - during the lockdown period, and $X_{1}$ to $X_{N}$ are the independent regression covariates. Since our focus is on how the pandemic lockdown affected their decisions, we only consider farmers who did not choose CRB in 2019 because if farmers used CRB in 2019 and used it again in 2020, the pandemic should not be a primary reason. In this analysis. We include a dummy variable on whether there was an associated delay in the removal of wheat residue because of it. Furthermore, we shall account for eight binary variables on how their lives were impacted during the pandemic – loss of employment, return migration to the village, loss of remittance, labor shortage, food shortage, having to sell assets, loss of agricultural income, and loss of other income source. The data from these responses are designed to shed more light on the farmers' decision-making processes in the pandemic's context. Controlling for these subjective assessments would help to identify the effect of the lockdown uniquely and more rigorously on farmers' choices of cleaner agricultural production methods. The analysis will also account for village fixed effects, respondent's awareness of the environmental effects of CRB, their perception of the effects of CRB on soil quality, and other socioeconomic covariates.

## 4 Results

### 4.1 Choice of wheat residue management

We begin our analysis by looking at the percentages of farmers who chose crop residue burning (CRB) during the postharvest wheat seasons during 2019 and 2020. We hypothesize that CRB occurs if the farmer's cost of removing wheat residue using a pro-environment (non-burning) method exceeds the tradable value of wheat residue. The results show that the use of CRB has increased from 2019 to 2020 from 12.93 to 17.53 percent, which is a 35.56 percent increase from 45 to 61 farmers.^4^ This difference is statistically significant (*p-value* < 0.05 using a one-sided *t*-test).

Our data show that 36 farmers switched their wheat residue management methods between the two years. Approximately 75 percent of these 36 farmers (26 farmers) changed their wheat residue management from non-CRB to the CRB method, while only ten farmers changed from CRB to non-CRB alternatives. These results indicate that CRB became more prevalent during the pandemic lockdown in Karnal, Haryana state. Wheat residue burning likely increased in Karnal, even though there are government regulations –such as fines– that are meant to reduce its occurrence [32,45].

### 4.2 Reasons of wheat residue burning

We examine a hierarchy of reasons behind wheat residue burning by asking farmers to list and rank them while keeping in mind the postharvest season of 2020. Table 1 shows that the severe shortage or unavailability of hired migrant labor and its consequent high cost are chosen by more than 90 percent of the farmers as a reason for wheat residue burning. More than half of the farmers explicitly state that residue burning was associated with the pandemic lockdown. Other reasons for residue burning, such as the high cost of hiring machines that collect residue, the use of combine harvesters, and no other use for residue are selected by approximately 25, 15, and 7 percent of the respondents, respectively.

**Table 1:** Ranking of reasons for wheat residue burning during 2020.

*Why did you choose to burn wheat residue? (N = 61)*		
**Reasons**	**Chosen by farmers**	**Chosen as most important reason**
Labor unavailable (*due to COVID lockdown*)	90.16%	50.82%
Expensive to hire labor	90.16%	37.70%
COVID lockdown	50.82%	4.92%
Expensive to hire machine	24.59%	3.28%
Combine harvester	14.75%	1.64%
No other use for residue	6.56%	1.64%

The ranking of these reasons is also reported in Table 1. More than half of the farmer respondents rank the unavailability of labor as the most important reason for wheat residue burning, followed by 38 percent who attribute it mostly to expensive labor costs. About 5 percent of farmer respondents state that the COVID-19 lockdown was the most important reason. Once again, we note that 90 percent of the farmers associate wheat residue burning with labor unavailability and its high cost during the lockdown. The other reasons – relating to hiring machines, combine harvesters, and no residual use – being ranked as most important are chosen by only a handful of the respondents. These results point to the COVID-19 lockdown leading to increased use of CRB among farmers; mostly, this is attributed to the shortage of hired farm labor during the nationwide lockdown.

Furthermore, we ask farmers who did *not* use CRB to lend their perspectives on why other farmers choose CRB in the postharvest wheat season. These results are reported in Table 2. Quite remarkably, nearly two-thirds of these farmers – who reportedly avoid burning wheat residue – have the perception that no farmer would rationally choose to burn wheat residue. They associate wheat residue as having tradable value and thus do not think any farmer would or should burn it. Our data indicate quite the opposite and thereby lower the credibility of such farmers’ perceptions. The use of CRB during the wheat postharvest might well be on the rise in Haryana, and the pandemic lockdown appears to have exacerbated this trend. Other farmers provided reasons similar to the reported reasons provided by those who chose CRB; however, the former appears to underestimate the impact of the lockdown – as suggested by 2.79 percent of them.

**Table 2:** Farmers’ perceptions on why other farmers choose CRB.

*What do you think is the most important factor why other farmers chose to burn wheat residue this year? (N = 287, for non-burners)*	
**Reasons**	**Percentage of farmers**
No one chooses to burn wheat residue	62.72%
Expensive to hire labor	16.03%
Expensive to hire machine	5.92%
Labor unavailable (due to COVID)	5.23%
Combine harvester	5.23%
COVID lockdown	2.79%
No other use for residue	2.09%

### 4.3 Alternative methods to manage wheat residue

If one chooses not to burn crop residue, then a natural question that follows would be: what are the alternative methods of crop residue management? In Table 3, we list the most popular alternative residue management methods and ask the farmers about their chosen options. We further ask them to rank their most preferred alternative to residue burning. Close to 80 percent of farmers use wheat residue as fodder for cattle, while about 40 percent of them choose to sell it. More than half of them reincorporate wheat residue into the soil, while two farmers gave away the residue freely to other farmers. The results clearly show that wheat residue is indeed highly valued by farmers, especially when compared with postharvest rice (paddy) residue; this concurs with our previous findings in Ref. [4], wherein several farmers report burning rice residue because it is unusable for anything else.

**Table 3:** Alternative methods for wheat residue management (other than CRB).

*When you do not burn wheat residue, what do you do with the residue? (N = 348)*		
**Options**	**Chosen by farmers**	**Chosen as most preferred option**
Fodder for cattle	79.31%	70.69%
Sell the residue	39.37%	21.26%
Reincorporate into soil	50.86%	8.05%
Give to others for free	0.57%	0%

Table 3 also lists the most preferred method to manage wheat residue without burning. More than two-thirds of the farmers state cattle fodder as their most preferred option, more than 20 percent end up trading it, and about 8 percent prefer to reincorporate wheat residue into the soil. The results imply that wheat residue is used mainly as fodder by farmers who raise cattle, and the remainder is sold by those who do not raise cattle or have a surplus of residue.

## 5 Analyzing the lockdown effect on CRB

In the previous section, we have shown that more farmers chose crop residue burning (CRB) during the postharvest wheat seasons in 2020 than they did during 2019. In this section, we explore the aspects or attributes of the pandemic that might have contributed to an increase in CRB. We focus our analysis on farmers who did not choose CRB in 2019 because if farmers used CRB in 2019 and used it again in 2020, the pandemic should not be a primary reason. Since the impacts of the pandemic differed among farmers, we will also explore and control for which types of impacts led to the use of CRB.

### 5.1 Descriptive statistics

In Table 4, we show that among 303 farmers who did not use CRB in 2019, 26 (8.58 percent) of them used CRB the year after. We compare their responses on whether there was a delay in the removal of wheat residue and sort by 8 particular impacts of the pandemic amongst the two groups: those who used CRB and those who did not use CRB in 2020. More than 80 percent of farmers who used CRB in 2020 reported a delay in the removal of wheat residue; this percentage is slightly over 40 percent among farmers who did not use CRB. This difference is statistically significant (*p*-value <0.001), which suggests that when farmers have less time to remove residue, they are more likely to use CRB since it is the quickest method to use.

**Table 4:** Attributes of the COVID-19 lockdown by residue management method in 2020.

*Did you burn wheat residue this year (2020)*?	Yes	No	(*p*-value)^a^
Number (percentage) of respondents^b^	26 (8.58%)	277 (91.42%)	–
Delay in wheat residue removal (%)	80.77	41.88	0.000***
**Attributes of COVID-19**:			
(a) Loss of employment (%)	7.69	23.83	0.060*
(b) Return migration to village (%)	7.69	10.11	0.695
(c) Loss of remittance (%)	7.69	12.27	0.492
(d) Labor shortage (%)	69.23	32.49	0.000***
(e) Food shortage (%)	15.38	18.05	0.735
(f) Having to sell assets (%)	15.38	8.66	0.259
(g) Loss of agricultural income (%)	96.15	58.48	0.000***
(h) Loss of other income source (%)	38.46	35.14	0.736

a *p*-value is two-sided. *, **, *** indicate error levels of 10%, 5%, and 1%, respectively.

b We only include farmers who did not choose CRB in 2019 so as to identify the lockdown effect.

Our survey contained a unique question designed to capture the respondent's subjective assessments of the lockdown effects on their lives in general. This allows us to delve deeper into the extraneous psychological aspects of farmers' decision-making in the context of the pandemic. Each farmer was asked this question: “How has your life changed during the COVID-19 pandemic?” Eight attributes or impacts were identified to them: (a) loss of employment, (b) return migration to village, (c) loss of remittances, (d) labor shortage, (e) food shortage, (f) having to sell assets, (g) loss of agricultural income, and (h) loss of other income source. The respondents could list any number of these attributes or impacts of the pandemic in response to the above question. Almost all farmers listed between one and four attributes in response; one farmer listed none of them, and another farmer listed seven of these attributes.

According to Table 4, we find that in attributes (a), (b), (c), and (e), there is a higher proportion of respondents choosing these in the group of farmers who did not use CRB in 2020. The difference between the two groups in attribute (a), loss of employment (23.83 versus 7.69 percent), is significant at the 10 percent error level (i.e., *p*-value = 0.060), while the other three attributes (b), (c), and (e) – return migration to the village, loss of remittance, and food shortage – are not statistically significant. For the remaining four attributes (d), (f), (g), and (h), a higher percentage of farmers who used CRB in 2020 were affected than those who did not use CRB. The differences in two attributes, (f) having to sell assets (15.38 versus 8.66 percent) and (h) loss of other income (38.46 versus 35.14 percent) are not statistically significant. However, differences in the other two attributes, (d) labor shortage (69.23 versus 32.49 percent) and (g) loss of agricultural income (96.15 versus 58.48 percent), are highly statistically significant (*p*-value <0.001). These two attributes – (d) and (g) – are closely related to farmers’ postharvest decisions and would likely increase the chances of using CRB for wheat residue management.

### 5.2 Regression analysis and results

Let us further investigate the factors influencing farmers' CRB decisions and examine if they corroborate the analysis with descriptive statistics. As outlined earlier in Section 3.2, we estimate a logit regression model given in Equation [1].^5^ The eight binary variables on how the farmers’ lives were impacted during the pandemic are listed as (a)-(h) in Table 5. These responses factor into the analysis as control variables. We will report two models that also include village fixed effects. In addition, Model [2] also includes other control variables like awareness of the environmental effects of CRB, perception of CRB effects on soil quality, and socioeconomic covariates – education, land size, house type, and farm assets.^6^

**Table 5:** Regression Coefficients of Determinants of Wheat Residue Burning Using Logit, (Binary dependent variable: choosing CRB in 2020 – Yes = 1, No = 0).

Variable	[1]	[2]
Delay in Wheat Residue Removal	1.70** (0.70)	1.78** (0.75)
**Attributes of COVID-19**:		
(a) Loss of employment	−0.14 (1.23)	0.11 (1.23)
(b) Return migration to village	−0.07 (0.69)	−0.02 (0.68)
(c) Loss of remittance	0.00 (0.57)	0.44 (0.45)
(d) Labor shortage	0.84* (0.49)	0.88* (0.50)
(e) Food shortage	0.87 (0.92)	0.77 (0.96)
(f) Having to sell assets	2.86** (1.24)	2.90** (1.25)
(g) Loss of agricultural income	3.28* (1.77)	3.47** (1.76)
(h) Loss of other income source	0.20 (0.41)	0.25 (0.39)
Include other variables	No	Yes
Pseudo R-Squared	0.234	0.248
Number of Observations	302	302

1. *, **, *** indicate error levels of 10%, 5%, and 1%, respectively.

2Robust standard errors clustered by village are in parentheses.

3All models include a constant and village fixed effects.

4We only include farmers who did not use CRB in 2019.

5Other variables in Model [2] are their awareness of the environmental effects of CRB, their perception of the effects of CRB on soil quality, education, land size, house type, and farm assets.

In Table 5, we report two logit regression results using Equation [1], in which the dependent variable is whether a farmer chose CRB in 2020, the lockdown year. Both models in Table 6 indicate that farmers who reportedly faced delays in removing wheat residue were more likely to choose CRB in 2020 (*p*-value <0.05). Among the pandemic attributes or impacts, (a)-(h), we observe three of them statistically increase the likelihood of CRB at either the 10 or 5 percent significance level. The three statistically significant impacts of the pandemic are (d) labor shortage, (f) having to sell assets, and (g) loss of agricultural income. A respondent's selection of attribute (f) implies a significant financial loss to them, and selecting the other two attributes, (d) and (g), points to a direct relationship with their farming activities. These results concur with our descriptive statistics discussed earlier in Section 5.1.

**Table 6:** Average marginal effects on wheat residue burning.

Independent Variable	Marginal Effects	
	Model [1]	Model [2]
Delay in Wheat Residue Removal	0.112*** (0.042)	0.116*** (0.043)
**Attributes of COVID-19**:		
(a) Loss of employment	−0.009 (0.082)	0.007 (0.080)
(b) Return migration to village	−0.005 (0.046)	−0.001 (0.044)
(c) Loss of remittance	0.000 (0.038)	0.028 (0.031)
(d) Labor shortage	0.055 (0.037)	0.058 (0.036)
(e) Food shortage	0.058 (0.062)	0.050 (0.063)
(f) Having to sell assets	0.190** (0.082)	0.189** (0.078)
(g) Loss of agricultural income	0.217* (0.116)	0.226** (0.112)
(h) Loss of other income source	0.013 (0.027)	0.016 (0.024)

1. *, **, *** indicate error levels of 10%, 5%, and 1%, respectively.

2Robust standard errors clustered by village are in parentheses.

Among the other control variables used in Model [2] in Table 5, Lopes et al. [4] report that many of these variables significantly affect farmers’ CRB decisions in rice residue management.^7^ Since we include only farmers who did *not* use CRB in 2019 and these control variables are common between the two years, we expect these variables not to significantly affect their CRB decisions in 2020. In other words, we should only observe variables that differ in 2020 from 2019 to have any effects on their decisions.^8^ The results in Model [2] show that all but one of the additional control variables do not significantly affect their burning decisions in this regard. The exception is the perception that CRB diminishes soil quality (*p*-value <0.05), and the sign is positive. In other words, farmers who believe that CRB diminishes soil quality were more likely to choose CRB.

Following the logit regressions in Models [1,2] from Table 5, we report the resulting average marginal effects in Table 6. First, we observe that farmers who faced a delay in the removal of wheat residue were significantly more likely to choose CRB in 2020 (*p*-value <0.01), i.e., by an increase in likelihood between 11.2 and 11.6 percent. Among eight attributes of the pandemic, only two of them significantly affect their CRB decisions. Those who had lost agricultural income (impact (g)) were 21.7–22.6 percent more likely to choose CRB (*p*-value <0.10 for Model [1] and *p*-value <0.05 for Model [2]), and those who had to sell assets because of the pandemic (impact (f)) were 18.9–19 percent more likely to choose CRB (*p*-value <0.05).

## 6 Discussion and conclusion

Postharvest crop residue burning (CRB) is a major policy concern for healthy agricultural ecosystems and environmental sustainability in several developing countries because it worsens air quality and reduces soil nutrients. Each year, the smoke from crop fires adds to the extant pollution in northern India's rural regions and neighboring urban centers with little to no success in lowering its occurrence. This paper explores how CRB was affected during the COVID-19 lockdown that coincided with the wheat harvesting season in northern India. The literature on the effects of COVID-19 lockdowns largely suggests that the reductions in economic activities led to beneficial effects on the environment. However, this narrative needs to be balanced with investigations into conceivably counterintuitive environmental outcomes of lockdowns. There is a lack of consensus in the literature on the effects of COVID-19 lockdowns being entirely beneficial to the environment. As noted in the Literature Review section earlier, it becomes crucial to investigate such matters further, particularly in developing countries. Such investigations are also important from a policy standpoint because resorting to lockdowns may not always benefit the environment. Public policy should bear in mind that the lack of monitoring and enforcement during lockdown periods would disincentivize pro-environmental choices. Governments should exercise caution when resorting to lockdowns in future pandemics. Strict control in trying to stop the spread of a pandemic disease by imposing nationwide lockdowns needs to be weighed against the likelihood of an increase in environmentally unfriendly behavior.

The case study explored in this paper contributes to the literature on the unintentional and perverse consequences of COVID-19 lockdowns - specifically in terms of its effects on CRB decisions in a developing country. We have presented substantial evidence of CRB increasing during the lockdown period in northern India. We collected and examined an original primary panel of crop residue management practices of 400 wheat farmers in Karnal, Haryana state, over two consecutive harvesting seasons of 2019 and 2020; the latter coinciding with the nationwide lockdown in India. We recorded these wheat farmers’ choices of postharvest residue management, which included CRB and non-CRB methods. We hypothesize that CRB occurs if the cost of removing crop residue using a pro-environmental method exceeds its tradable value. A salient finding is that more farmers burned their postharvest wheat residue during the nationwide lockdown in 2020 than in the previous year. More than 90 percent of the farmers identified labor shortage, the unavailability of hired labor, and its consequent high cost as the main reasons for wheat residue burning. The nationwide lockdown blocked the free movement of farm labor, thereby leading to acute labor shortages and substantial increases in hired labor costs. With higher costs to remove wheat residue, many farmers were seemingly driven to burn the residue instead of choosing to remove the bulk of it for cattle fodder or trading in it. Our findings suggest a prevalent perception amongst the farmers that the benefits of wheat residue did not exceed its removal costs during the lockdown period. In 2019, about 13 percent of farmers burned postharvest wheat residue; this increased to about 18 percent in 2020, yielding a statistically significant difference.

The novelty and strength of our paper lies in the collection and examination of unique and original panel data of CRB choices over two harvesting seasons in northern India – during the pre-lockdown and lockdown phases. To our knowledge, this study is also the first to examine CRB practices over these two timeframes in the Indian context. We find that wheat residue burning witnessed a significant increase between these two years – even though it retains value as cattle fodder. There is a 36 percent increase in the number of farmers who reportedly chose CRB for wheat residue management in 2020. Our survey expounds the main reason as a sudden stop to farm labor mobility during the nationwide lockdown. With the COVID-19 pandemic lockdown imposed nationwide across India during March–May of 2020, the issue of wheat residue burning appears to have unintentionally worsened.

Several studies compare economic decisions in pre-lockdown and during-lockdown phases. We noted how those studies have found both positive and negative effects of COVID-19 lockdowns on economic decisions and environmental outcomes – such as stoppages to economic activity leading to reductions in pollutants like sulfur dioxide, carbon monoxide, and particulate matter, and conversely to an increase in these and other pollutants like ozone in different geographical locations and atmospheric altitudes. Even though our approach is similar to other studies in the literature in terms of the timeframe of comparison, we have focused on the effects of the COVID-19 lockdown on economic decision-making surrounding CRB. Moreover, we have provided a unique perspective by accounting for farmers’ subjective opinions on socioeconomic attributes of the COVID-19 lockdown and examining their effects on CRB choices.

The pandemic and resulting nationwide lockdown disrupted the agrarian sector in India. This paper is the first to examine how the impacts of the pandemic may have contributed to the reported increase in CRB. Considering that the pandemic's impacts differ among farmers, we have explored and controlled for its various attributes that may have led to the use of CRB. We included a unique survey question to capture the subjective assessments of farmers on how the lockdown affected their lives in general. Each farmer was asked the question “How has your life changed during the COVID-19 pandemic?” in regard to eight attributes: (a) loss of employment, (b) return migration to village, (c) loss of remittances, (d) labor shortage, (e) food shortage, (f) having to sell assets, (g) loss of agricultural income, and (h) loss of other income source. Respondents could choose multiple attributes, and we could analyze their relative importance. We have used their responses to derive unique insights into farmers' decision-making processes in the context of the pandemic and lockdown. The sudden shock to labor mobility during the lockdown has thus been accounted for. Reduced labor mobility was associated with environmentally friendly farmland management options becoming more expensive in India.

We relied on binary variable regression techniques to assess how the lockdown affected CRB decisions while controlling for farmer heterogeneity in socioeconomic characteristics and the attributes of the pandemic. Significant evidence supports the contention that farmers who faced delays in removing wheat residue were more likely to choose CRB during the lockdown. Three of the pandemic's attributes or impacts significantly increased the likelihood of CRB – (d) labor shortage, (f) having to sell assets, and (g) loss of agricultural income. The selection of attribute (f) tells a story of significant financial loss to the farmers resulting from the pandemic. The other two attributes point to how their farming activities were adversely impacted by the lockdown. It appears that CRB is a rational response of several farmers in exigent circumstances. Noteworthy among the marginal effects, derived from the binary regressions, was that farmers were nearly 12 percent more likely to choose CRB in 2020 because of the lockdown delays in wheat residue removal. Furthermore, attributable to the pandemic and resulting lockdown, those who had lost agricultural income were 23 percent more likely to choose CRB, and those who were forced to sell assets were up to 19 percent more likely to choose CRB. Controlling for the respondents' subjective assessments helps to uniquely and more rigorously identify the nuances of the lockdown effects on farmers' choices of environmentally friendly postharvest methods.

We are mindful that a limitation of this study lies in the size of our sample of 400 farmers in northern India. The budgetary limitations for this study dictated a survey of a small number of sampled farmers' responses. This allows us to extrapolate our findings only cautiously to other region's agricultural systems. Nevertheless, the lessons from this paper are plausibly relevant not only to northern India's states but to other countries that are subject to the CRB issue in general. More research in this area is called for in order to better understand the effects of lockdowns, should such pandemics arise in the future. In order to discourage CRB as a go-to choice by farmers, policy design needs to cater to either increasing the benefits of non-CRB methods or decreasing their costs. A feasible avenue to address wheat residue burning is to facilitate market access for tradable residue since it already commands economic value and is traded informally by farmers in the region. Unburnt crop residue has the potential to lead to enhanced economic value in the wider economy. For instance, Härri et al. [38] examine how farmers in India can supply agricultural residue for the production of fibers in a circular and sustainable textile market system. Similarly, Jusakulvijit et al. [46] report how residues from Thailand's sugarcane and rice crops can feed into the production of economically valuable bioethanol.

Market-oriented policies for tradable crop residue could go a long way in reducing the practice of burning it. Another avenue would include the reduction in cost for farmers via state government lending or the subsidizing of farm machinery that can automatically remove postharvest residue.^9^ Our primary data indicate that farmers in the region perceive this option as being rather costly from their private perspectives since they have to bear its costs. In order to increase the benefits of pro-environment residue management for farmers, state and national governments should facilitate a platform that enables wider and more penetrating market access for tradable wheat residue. If farmers can easily remove the wheat residue from their farms without burning it and be guaranteed an avenue to sell any surplus on the market, the number of farmers who opt for CRB would potentially decline.

In a previous study, we found that more farmers choose CRB if they perceive others as also adopting it [4]. Conversely, if fewer farmers use CRB then others might plausibly follow suit. This points to the importance of social conformity and the role that societal norms can play in encouraging socially desirable or pro-environmental behavior [47,48]. Careful examination and identification of the behavioral aspects of agricultural and environmental choices are crucial to policy success. This study contributes to a growing literature on the ambiguous and unintended behavioral effects of lockdowns in the context of an important policy issue for developing agrarian economies.

## Funding statement

We greatly appreciate the financial support from the American University of Sharjah (Grant numbers FRG20-S-B79 and OAPSBA-1210-B00018). This paper represents the opinions of the authors and does not mean to represent the position or opinions of the American University of Sharjah.

## Data availability statement

Data will be made available on request.

## Additional information

The authors thank Probe and Search India for their professional phone survey work and Soumya Gupta for her insightful comments. Supplementary contents related to this article has been published online.

## CRediT authorship contribution statement

**Adrian A. Lopes:** Writing – review & editing, Writing – original draft, Methodology, Funding acquisition, Conceptualization. **Ajalavat Viriyavipart:** Writing – review & editing, Writing – original draft, Methodology, Formal analysis, Conceptualization.

## Declaration of competing interest

The authors declare that they have no known competing financial interests or personal relationships that could have appeared to influence the work reported in this paper.

## Footnotes

1 Lopes et al. [40] show that farmers who burn their rice residue are more likely to burn wheat residue in a subsequent cycle.

2 There was a farmer union rule in one village where farmers who were caught burning their wheat residue would be sanctioned; thereby we excluded the farmers from that village from our final analysis. We also excluded two farmers from one village who were not willing to answer whether they had burned wheat residue; this leaves us with 348 subjects from the remaining seven villages. The budgetary limitations of the study only permitted surveying a sample of this size.

3 Details on each village about the number of households, the number of paddy farmers, the number of paddy farmers surveyed in Lopes et al. [4], and the number of wheat farmers surveyed in this study are provided in Table S1 in the appendix.

4 As noted earlier, we excluded two farmers from Village 6 who were not willing to answer whether they had burned wheat residue.

5 A probit regression provides similar results; we have decided to report the results based on only the logit model.

6 The regression excludes one perception related to whether CRB is a common practice in the village because of perfect multicollinearity; none of the farmers who responded that CRB is not a common practice at all (choice 1) chose CRB.

7 Lopes et al. [4] show that farmers who [1] think that CRB is a common practice [2], think that CRB diminishes soil quality [3], have a larger size of cultivated land, and [4] have better quality houses, are more likely to use CRB.

8 As we have shown earlier, these variables are the delay in the removal of wheat residue and three attributes of the pandemic.

9 For example, Lopes et al. [3] show in a field experiment that conditional cash transfers to farmers who use environmentally-friendly residue management methods can go a long way in lowering the occurrence of CRB.
