# Supplementary material for: Crop residue burning increased during the COVID-19 lockdown: A case study of rural India
Source: Heliyon. 2024 Mar 9;10(6):e27910. doi: 10.1016/j.heliyon.2024.e27910 (PMC10950696; doi:10.1016/j.heliyon.2024.e27910)
Supplement: Multimedia component 1 [file mmc1.docx]

**APPENDIX**

**Table S1: Number of households, number of paddy farmers, number of paddy farmers** **surveyed in Lopes et al. (2020), and number of wheat farmers surveyed in this study.**

| Village Number (as in Lopes et al., 2020) | Number of Households^1^ | Number of Paddy farmers^1^ | Number of paddy farmers in Lopes et al. (2020) | Number of wheat farmers in this study |
| --- | --- | --- | --- | --- |
| 1 | 625 | 450 | 98 | 50 |
| 2^2^ | 850 | 650 | 108 | 50 |
| 3 | 300 | 175 | 92 | 50 |
| 4 | 330 | 200 | 112 | 50 |
| 5 | 750 | 350 | 99 | 0 |
| 6 | 725 | 425 | 111 | 50 |
| 7 | 700 | 450 | 98 | 0 |
| 8 | 250 | 210 | 99 | 50 |
| 9 | 300 | 150 | 97 | 0 |
| 10 | 800 | 450 | 101 | 50 |
| 11 | 650 | 350 | 106 | 0 |
| 12 | 1900 | 1100 | 109 | 50 |
| **Total** | **8180** | **4960** | **1230** | **400** |

Notes:

1) The numbers of households and paddy farmers are based on information collected during November 2019 from the elected heads of each village.

2) There was a farmer union rule in Village 2 where farmers who were caught burning their wheat residue would be sanctioned; thereby we excluded them from our final analysis.

**Survey Questions**

| Date of the interview |  |  | 2020 | Date checked |  |  | 2020 |  |
| --- | --- | --- | --- | --- | --- | --- | --- | --- |
| Signature of enumerator: |  | | | Signature of supervisor: |  | | |  |
| Time started: |  | | | Time ended: |  | | |  |
| Village name: गांव का नाम | , Karnal | | | Household ID#: | (confirm same ID# per original survey) | | |  |

1. **Household Composition and Farm Characteristics**

| 1.1 | Father’s name  पिता का नाम | (confirm same father name per original survey) (पहले से उपलब्ध डाटा से पिता का नाम जांचें) | | | | | |
| --- | --- | --- | --- | --- | --- | --- | --- |
| 1.2 | Respondent Mobile no.  उत्तरदाता का मोबाइल नंबर |  | | | | | |
| 1.3 | Relationship of respondent with household head  घर के मुखिया के साथ उत्तरदाता का रिश्ता |  | | | | | |
|  | | 1.4.1  Name नाम | 1.4.2 Gender लिंग  Code A | 1.4.3 Age  Years आयु (वर्ष) | 1.4.4 Education शिक्षा  Code B | 1.4.5  Primary job प्राथमिक व्यवसाय  Code C | 1.4.6 Secondary job दूसरा व्यवसाय  Code C |
| 1.4 Respondent (*person making farming decision*) उत्तरदाता (जो खेती का निर्णय करता है) | |  |  |  |  |  |  |

|  | Land units खेती की भूमि  (Int: कनाल को किल्ले में बदलें) | *Cultivated खेती की गयी*  *(किल्ला या कनाल)* | *Owned कुल जमीन*  *(किल्ला या कनाल)* |
| --- | --- | --- | --- |
| 1.5 | Land units for wheat in 2020  2020 में गेहूं के लिए खेती की भूमि |  |  |
| 1.6 | Land units for other crops during wheat season in 2020  गेहूं के मौसम (रबी) में 2020 में अन्य फसलों के लिए खेती की भूमि |  |  |
| **Code A:** 1 – Maleपुरुष; 2 – Female महिला  **Code B**: 0–No education/illiterate कोई शिक्षा नहीं / अशिक्षित; 1–semi-literate (never attended school) अर्द्ध साक्षर; 2 –Primary (Grade 1-5 प्राथमिक (ग्रेड 1-5); 3– Middle (Grade 6-8) पूर्व माध्यमिक (ग्रेड 6-8); 4 – Secondary (Grade 9-10) माध्यमिक (ग्रेड 9-10); 5 – Higher Secondary (Grade 11-12) उच्चतर माध्यमिक (ग्रेड 11-12) ; 6– Graduate स्नातक; 7–Post graduate स्नातकोत्तर; 8–Vocational education व्यावसायिक शिक्षा  **Code C :**1– Farming खेती; 2 – Livestock rearing पशु पालन; 3 – Salaried employmentवेतनभोगी रोजगार; 4 – Self-employed off farm खेत के अलग अन्य स्वरोजगार,; 5 – Casual labourer on farm कृषि मज़दूर; 6 – Casual labourer off farm गैर कृषि मज़दूर ; 7 – Involved in household chores घर के काम में शामिल; 99 – Other (specify) अन्य (लिखें) | | | |

1. **Crop Residue Management**

| 2.1 | Is burning wheat residue a common practice in your village?  क्या आपके गांव में गेहूं (कनक) के अवशेष (पराली) को जलाना एक आम बात है? | - Not at all बिल्कुल नहीं - Occasionally कभी-कभी - Often अक्सर - Very common बहुत ही आम - Everybody does it हर कोई यह करता है | | | 1  2  3  4  5 | | | |
| --- | --- | --- | --- | --- | --- | --- | --- | --- |
| 2.2 | What was the time period of wheat residue removal for you in April/May 2020?  अप्रैल/मई 2020 में आपने गेहूं (कनक) की पराली किस तिथि से किस तिथि तक हटाया ? | Start date शुरू होने की तिथि | | |  | | | |
|  |  | End date समाप्त होने की तिथि | | |  | | | |
| 2.3 | Was there a delay in removal of wheat residue this year compared to last year?  क्या पिछले साल की तुलना में इस साल गेहूं (कनक) के पराली को हटाने में **देरी** हुई? | Yes/ No/ Unwilling to Answer  (1/ 0 /99)  हां / नहीं / जवाब नहीं देना चाहते  (1/ 0 /99) | | | | | | |
| 2.4 | If YES to 2.3 – Did the Covid lockdown cause this delay in residue removal?  यदि 2.3 में हाँ है तो पूछें- क्या पराली को हटाने में हुयी इस देरी का कारण "**कोविड लॉकडाउन**" था? | Yes/ No/ Unwilling to Answer  (1/ 0 /99)  हां / नहीं / जवाब नहीं देना चाहते  (1/ 0 /99) | | | | | | |
| 2.5 | If YES to 2.3 – Did the lack of migratory labour cause this delay in residue removal?  यदि 2.3 में हाँ है तो पूछें- क्या पराली को हटाने में हुयी इस देरी का कारण "**प्रवासी श्रमिकों की कमी"** थी? | Yes/ No/ Unwilling to Answer  (1/ 0 /99)  हां / नहीं / जवाब नहीं देना चाहते  (1/ 0 /99) | | | | | | |
| 2.6 | Did you burn wheat residue on your land last year/season? क्या आपने पिछले साल / सीजन में अपनी भूमि पर गेहूं (कनक) के पराली को जलाया था? | Yes/ No/ Unwilling to Answer  (1/ 0 /99)  हां / नहीं / जवाब नहीं देना चाहते  (1/ 0 /99) | | | | | | |
| 2.7 | Did you burn wheat residue on your land this year/season? क्या आपने इस साल / सीजन में अपनी भूमि पर गेहूं (कनक) के पराली को जलाया था? | Yes/ No/ Unwilling to Answer  (1/ 0 /99)  हां / नहीं / जवाब नहीं देना चाहते  (1/ 0 /99) | | | | | | |
| 2.8 | If YES to 2.7, what were the important factors why you chose to burn wheat residue?  यदि 2.7 में हाँ है तो पूछें- वे कौन-कौन से महत्वपूर्ण कारण थे, जिनकी जिसकी वजह से आपने गेहूं (कनक) के पराली को जलाया? | Covid lockdown कोविड लॉकडाउन | | | | 1 | | |
|  |  | Labor unavailable due to Covid lockdown. कोविड लॉकडाउन के कारण श्रमिकों की कमी | | | | 2 | | |
|  |  | Expensive to hire labor महंगा श्रम | | | | 3 | | |
|  |  | No other use for residue  अवशेष का कोई अन्य उपयोग नहीं | | | | 4 | | |
|  |  | Combine harvester कम्बाइन हारवेस्टर के कारण | | | | 5 | | |
|  |  | Expensive to hire machinery  मशीन का महंगा किराया | | | | 6 | | |
|  |  | Other (specify): अन्य कारण (लिखें) | | | | 7 | | |
| 2.8.1 | And what was the most important reason among these? Single answer  और इनमें से एक सबसे महत्वपूर्ण वजह क्या थी? | One Code from Q 2.8  Q 2.8 से एक कोड करें | | | |  | | |
| 2.9 | If YES to 2.7, did the Covid lockdown cause you to burn wheat residue?  (Enumerator: ask only if respondent did not choose option 1 or 2 in Q 2.8)  यदि 2.7 में हाँ है तो पूछें- क्या कोविड लॉकडाउन के कारण आपने गेहूं (कनक) के पराली को जलाया?  (प्रश्नकर्ता: केवल तभी पूछें जब उत्तरदाता ने Q 2.8 में विकल्प 1 या 2 नहीं चुना है) | Yes/ No/ Unwilling to Answer  (1/ 0 /99)  हां / नहीं / जवाब नहीं देना चाहते  (1/ 0 /99) | | | | | | |
| 2.10 | If YES to 2.9, explain reason:  यदि 2.9 में हाँ है तो, **कारण** का वर्णन करें: |  | | | | | | |
| 2.11 | (Ask all), what do you think is the most important factor why other farmers chose to burn wheat residue this year?  सभी से पूछें- आपके अनुसार, इस वर्ष किसानो द्वारा गेहूं (कनक) के पराली को जलाने का मुख्य कारण क्या थे? | Covid lockdown कोविड लॉकडाउन | | | | | 1 | |
|  |  | Labor unavailable due to Covid lockdown. कोविड लॉकडाउन के कारण श्रमिकों की कमी | | | | | 2 | |
|  |  | Expensive to hire labor महंगा श्रम | | | | | 3 | |
|  |  | No other use for residue  अवशेष का कोई अन्य उपयोग नहीं | | | | | 4 | |
|  |  | Combine harvester कम्बाइन हारवेस्टर के कारण | | | | | 5 | |
|  |  | Expensive to hire machinery  मशीन का महंगा किराया | | | | | 6 | |
|  |  | No one burned किसी ने नहीं जलाया | | | | | 7 | |
|  |  | Other (specify): अन्य कारण (लिखें) | | | | | 8 | |
| 2.12 | When you do not burn wheat residue, what do you do with the residue? (Only for this year)  जब आप गेहूं (कनक) के अवशेष (पराली) को नहीं जलाते हैं तो आप उस अवशेष (पराली) का क्या करते हैं? (इस वर्ष के लिए) | Reincorporate into soil मिट्टी में दबा देते हैं | | | | | 1 | |
|  |  | Sell the residue पराली को बेच देते हैं | | | | | 2 | |
|  |  | Fodder for cattle जानवरों के चारे के रूप में इस्तेमाल करते है | | | | | 3 | |
|  |  | Make residue briquettes पराली का ब्रिकेट (ठोस बण्डल) बनाते हैं | | | | | 4 | |
|  |  | Other (specify): अन्य (लिखें) : | | | | | 5 | |
| 2.12.1 | When you did not burn wheat residue this year, what was your most preferred option for managing it? (select one from Q2.12)  यदि आपने इस वर्ष गेहूं की पराली को नहीं जलाया तो आपने पराली का सबसे ज्यादा क्या किया? (Q2.12 में बताये गए जवाबों में से एक चुने) | One Code from Q 2.12  Q 2.12 से एक कोड करें | | | | |  | |
| 2.13 | Did the Covid lockdown cause you to change your wheat residue burning decision?  क्या कोविड लॉकडाउन की वजह से, गेहूं (कनक) के पराली को जलाने से सम्बंधित आपके फैसले पर कोई असर हुआ? | Don’t usually burn but did burn this year  आमतौर पर नहीं जलाते लेकिन इस साल जलाया | | | | | 1 | |
|  |  | Usually burn but didn’t do so this year आमतौर पर जलाते हैं लेकिन इस साल ऐसा नहीं किया | | | | | 2 | |
|  |  | No change in decision to burn  जलाने के निर्णय में कोई बदलाव नहीं | | | | | 3 | |
|  |  | Unwilling to Answer जवाब नहीं देना चाहते | | | | | 99 | |
| 2.14 | Are there any farmer unions in your village?  क्या आपके गाँव में कोई किसान यूनियन है? | Y/N (1/0)  (हाँ =1 , नहीं=0 ) | | | | | | |
| 2.15 | If YES to 2.14 – Did unions make any new rules for CRB this year?  यदि 2.14 में हाँ है तो पूछें-क्या यूनियन ने इस साल पराली जलाने से सम्बंधित कोई नया नियम बनाया है? | Y/N (1/0)  (हाँ =1 , नहीं=0 ) | | | | | | |
| 2.16 | If YES to 2.15, then what type of rules were they?  यदि 2.15 में हाँ है तो पूछें-यह नियम किस प्रकार का है? | Sanction/notification if caught burning.  यदि जलाते हुए पकड़े गए तो प्रतिबंध/ अधिसूचना | | | | 1 | | |
|  |  | Machine availability for residue removal.  अवशेष हटाने के लिए मशीन की उपलब्धता | | | | 2 | | |
|  |  | Cash subsidy for residue removal.  अवशेष हटाने के लिए नकद सब्सिडी | | | | 3 | | |
|  |  | Direct payment to farmer  किसान को सीधा भुगतान | | | | 4 | | |
|  |  | Other (specify): अन्य (लिखें): | | | | 5 | | |
| 2.17 | If YES to 2.15, did the new union rules reduce residue burning significantly?  यदि 2.15 में हाँ है तो पूछें-क्या यूनियन के इस नए नियम से, पराली जलाना काफी कम हुआ है? | Yes/ No/ DK or Unwilling to Answer  (1/ 0 /99)  हाँ/ नहीं / पता नहीं या जवाब नहीं देना चाहते  (1/ 0 /99) | | | | | | |
| 2.18 | Did the govt. come up with any specific programs during the Covid lockdown to assist farmers with wheat residue management?  क्या कोविड लॉकडाउन के दौरान, किसानों द्वारा गेहूं के पराली के प्रबंधन में सहायता के लिए सरकार कोई ख़ास योजना लाई है? | Y/N (1/0)  (हाँ =1 , नहीं=0 ) | | | | | | |
| 2.19 | If Yes to 2.18 – What type of program is it?  यदि 2.18 में हाँ है तो पूछें- यह किस प्रकार की योजना है? | Cash subsidy (conditional on not burning).  Describe:  नकद सब्सिडी (नहीं जलाने की शर्त के साथ)  वर्णन करें: | | | | 1 | | |
|  |  | Cash subsidy (non-conditional direct payment).  Describe:  नकद सब्सिडी (बिना शर्त के सीधा भुगतान)  वर्णन करें: | | | | 2 | | |
|  |  | Residue briquettes sale.  Describe:  पराली के ब्रिकेट (ठोस बण्डल) की बिक्री  वर्णन करें: | | | | 3 | | |
|  |  | Machine availability.  Describe:  मशीन की उपलब्धता  वर्णन करें: | | | | 4 | | |
|  |  | Other.  Describe:  अन्य  वर्णन करें: | | | | 5 | | |
|  | Estimate the time and cost of removing wheat residue **per acre** of land.  एक एकड़ (किल्ला) खेत से गेहूं की पराली को हटाने में में लगने वाली कीमत और समय का अंदाज़ा लगाएं |  | | *Last year:*  *पिछले साल* | *This year:*  *इस साल* | | | |
|  |  | 2.20 Total cost of machine removal **per acre** मशीन से 1 किल्ला अवशेष हटाने में कुल खर्च | |  |  | | | |
|  |  | 2.21 Total cost of labor removal **per acre** श्रमिक द्वारा 1 किल्ला अवशेष हटाने में कुल खर्च | |  |  | | | |
| 2.22 | Report the selling price of wheat residue per quintal (Leave blank if don’t know)  प्रति क्विंटल गेहूं की पराली का विक्रय मूल्य क्या है? (यदि पता नहीं है तो खाली छोड़ दें) |  | | *Last year:*  *पिछले साल* | *This year:*  *इस साल* | | | |
|  |  | Residue price per quintal प्रति क्विंटल पराली का मूल्य | |  |  | | | |
| 2.22A | What is the quantity of wheat residue generated per acre? प्रति एकड़ कनक की पराली की पैदावार क्या थी? | Per Acre in quintal प्रति एकड़ क्विंटल में | |  |  | | | |
| 2.22B | How many quintal of wheat residue you sold per acre?  उसमे से प्रति एकड़ कितनी पराली आपने बेची? | Per Acre in quintal प्रति एकड़ क्विंटल में | |  |  | | | |
| 2.23 | Was there a delay in wheat procurement by government this year?  क्या सरकार द्वारा इस वर्ष गेहूं खरीद में देरी हुई? | Y/N (1/0)  (हाँ =1 , नहीं=0 ) | | | | | | |
| 2.24 | If YES to 2.23 – Was delay because of the Covid lockdown?  यदि 2.23 में हाँ है तो पूछें- क्या कोविड लॉकडाउन की वजह से यह देरी हुई? | Yes/ No/ Unwilling to Answer  (1/ 0 /99)  हाँ/ नहीं / जवाब नहीं देना चाहते  (1/ 0 /99) | | | | | | |
| 2.25 | What was the MSP of wheat?  गेहूं का न्यूनतम समर्थन मूल्य क्या था ? | *Last year* (Rs. per quintal)*:*  *पिछले साल (रू.* प्रति क्विंटल) | *This year* (Rs. per quintal)*:*  *इस साल (रू.* प्रति क्विंटल) | | | | | |
|  |  |  |  | | | | | |
| 2.26 | How has your life changed during the COVID-19 pandemic?  कोविड-19 माहमारी की वजह से आपके जीवन पर क्या क्या प्रभाव पड़ा? | - Loss of employment रोजगार/व्यवसाय बंद होना | | | | | | 1 |
|  |  | - Return migration to village गाँव में वापसी | | | | | | 2 |
|  |  | - Loss of remittance गांव/ घर पैसे न भेज पाना | | | | | | 3 |
|  |  | - Labor shortage श्रमिकों की कमी | | | | | | 4 |
|  |  | - Food shortage भोजन की कमी | | | | | | 5 |
|  |  | - Had to sell assets सम्पत्तियों को बेचना पड़ा | | | | | | 6 |
|  |  | - Loss of agricultural income खेती से होने वाली आय का नुकसान | | | | | | 7 |
|  |  | -Loss of other income source अन्य श्रोतों से होने वाली आय का नुकसान  Specify loss of other income source  जिस स्रोत से नुक्सान हुआ उसका नाम: | | | | | | 8 |
|  |  | - other (Please specify) अन्य (लिखें) | | | | | | 9 |
| 2.26.1 | And what was the most severe impact of Covid-19?  और इनमे से कोविड-19 का सबसे गंभीर प्रभाव क्या था? | One Code from Q 2.26  Q 2.26 से एक कोड करें | | | | | |  |
